# Genealogy, Dendritic Cell Priming, and Differentiation of Tissue-Resident Memory CD8^+^ T Cells

**DOI:** 10.3389/fimmu.2018.01751

**Published:** 2018-07-31

**Authors:** Michel Enamorado, Sofía C. Khouili, Salvador Iborra, David Sancho

**Affiliations:** ^1^Centro Nacional de Investigaciones Cardiovasculares (CNIC), Madrid, Spain; ^2^Department of Immunology, School of Medicine, Universidad Complutense de Madrid, 12 de Octubre Health Research Institute (imas12), Madrid, Spain

**Keywords:** memory CD8^+^ T cell, circulating memory, tissue-resident memory, infection, plasticity, priming, differentiation, dendritic cells

## Abstract

Tissue-resident memory CD8^+^ T (Trm) cells define a distinct non-recirculating subset. Trm cells constitute a first line of defense against local infections in barrier tissues, but they are also found in non-barrier tissues and play a role in antitumor immunity. Their differentiation in tissues and their phenotypical, transcriptional, and functional characteristics are the object of active research. Herein, we will discuss the potential existence of committed CD8^+^ Trm precursors and the genealogy of memory CD8^+^ T cell subsets. In addition to the priming of naive T cells, there is some plasticity of antigen-experienced effector and memory T cell subsets to generate Trm precursors. Local inflammation, antigen presentation, and cytokines drive Trm differentiation. It is of prime interest how specific dendritic cell subsets modulate priming and differentiation of Trm cells, as well as their reactivation within tissues. Research on how we can manipulate generation of memory T cells subsets is key for improved vaccination strategies.

## Are There Committed Trm Precursors?

Dendritic cells (DCs) prime naive T cells in secondary lymphoid organs generating both a short-term effector response and a memory response. Memory T cells are further subdivided based on their distribution and trafficking properties. Circulating memory T cells can be further subdivided as central memory T (Tcm) cells that re-circulate between secondary lymphoid organs, blood and lymph, and effector memory T (Tem) cells that can also access the tissues (1, 2). Conversely, a distinct subset of sessile tissue-resident memory T (Trm) cells has been defined in the last years. Trm cells are long-lived and confined in a wide variety of tissues, including barrier tissues, such as the skin and lung, where they comprise the first line of defense against local re-infections and provide superior protective immunity compared with circulating memory cells (3–7). However, Trm cells are also found in non-barrier tissues like brain (8), heart (9), and play a role in tumor immunity (Box 1) (10–12). Trm cells are phenotypically, transcriptionally, and functionally distinct from their circulating counterparts. Trm cells do not express the lymph node homing receptors CCR7 or CD62L, and expression of CD69 and the integrin CD103 is often used to define T cells as tissue resident (7, 13). However, CD103^−^ CD69^+^ cells make up almost half of the dermal Trm population (3, 14), while the intestine and the lung contain subsets of T cells that lack CD103 and/or CD69 expression but are nonetheless capable of maintaining tissue residence (15–17). This phenotypic heterogeneity among Trm populations is dependent not only on the tissue of residence but also on how Trm cells are generated by local tissue infection. In addition, Trm cells exhibit a unique transcriptional signature that comprises modulation of chemokine receptors like CXCR3 (4), upregulation of genes associated to tissue residency including *Cdh1* (E-cadherin) (18), *Itgae* (CD103) (8, 19), and *Itga1* (CD49a) (13, 20–22), and downregulation of genes related to tissue egress, such as *Klf2, S1pr1* (23), and *Ccr7* (4, 24) among others. They also show augmented effector function compared with circulating memory cells, with elevated expression of *Granzyme B* and *Tnf-a*, and genes encoding immunoregulatory molecules such as ICOS and CTLA-4, indicating tight modulation of the robust effector function of Trm cells (4, 24). Importantly, this transcription core is shared between human and mouse Trm cells (25–27).

Box 1 **Trm in immunity against tumors.**

The relative contribution of different memory CD8^+^ T cell subsets to antitumor immunity is starting to be explored. Data in human tumors show that the number of cells with a Trm phenotype infiltrating tumors correlates with a better overall survival in different cancers, including early stage non-small-cell lung carcinoma, pulmonary squamous cell carcinoma, and high-grade serous epithelial ovarian cancer (11, 28–31). Immunotherapy of cancer using vaccination routes that generate Trm may be superior in generation of antitumor immunity (32, 33). In addition, reprogramming of infiltrating DCs in the tumor with curdlan induce a Trm phenotype in tumor-infiltrating T cells that can reject tumors (34). The contribution of Trm to cancer immunity has been explored in several mouse cancer models. Using a mouse model of melanoma-associated vitiligo induced by depletion of regulatory T cells and surgical excision of a primary dermal B16 melanoma, functional melanoma antigen-specific Trm cells develop in the skin of mice with vitiligo and are critical for protection against melanoma rechallenge (10). Intranasal vaccination with a mucosal vector targeting DCs fused to an HSV-derived peptide leads to generation of Trm that are protective against an orthotopic head and neck TC1 tumor (11). Following skin scarification with rVACV-OVA, both circulating memory CD8^+^ T cells and Trm cells are sufficient to mediate immunity against B16-OVA melanoma (12). Surgical parabiosis of rVACV-OVA skin-scarified mice with naive mice leads to share circulating memory T cells while antigen-specific Trm cells are restricted to the immunized parabiont. Challenge with melanoma of separate parabionts shows that circulating memory cells transfer antitumor immunity but this response is improved in the presence of Trm cells (12). In addition, Tcm cell infiltration in the tumor also induce the generation of cells with a Trm phenotype expressing PD-1, showing that anti-PD1 therapy can improve the effectiveness of Trm cells within the tumor (12). These results suggest that strategies aimed to enhance Trm generation or infiltration within tumors, in cooperation with circulating memory T cells, may result in improved cancer immunotherapy.

Trm cells can be generated from KLRG1^lo^ memory precursors (4). These KLRG1^lo^ memory precursors are either KLRG1^−^ IL-7Rα^+^ memory precursor CD8^+^ effector T cells or KLRG1^+^ effector cells that have lost KLRG1 expression (ExKLRG1) (35). These cells seed in non-lymphoid tissue where differential expression of transcription factors and tissue-derived signals instruct the tissue residency program of this T cell lineage. Trm formation requires partial downregulation of T-bet and complete shutdown of eomesodermin (Eomes), being both events controlled by TGF-β derived from the tissue. Remaining T-bet is critical for IL-15R expression, which allows responsiveness to tissue-derived IL-15 necessary for their long-term survival (36). T-bet along with IL-15 signaling are also critical for the expression of the transcription factor Hobit, that is essential for establishment of Trm cells in the tissue. Hobit cooperates with the transcription factor Blimp1 to control the transcriptional program of residency of Trm cells and concomitantly blocks the differentiation to alternative T cell memory lineages (37) and regulates effector functions in quiescent human effector-type CD8^+^ T cells (38). In addition, the transcription factor Runx3 is also a key regulator of Trm generation and modulates tissue residency (39).

Recent studies have revealed important contributors to Trm cell establishment and differentiation in the tissue (4–7, 23). However, less is known about the early priming signals in secondary lymphoid organs that precede entry into peripheral tissues (14). While both resident and circulating memory T cells have a common naive precursor (40), there are evidences suggesting the existence of a committed Trm precursor. Modulation of T cell metabolic reprogramming affects Trm generation acting early after activation and determining T cell fate and function (41). Specifically, inhibition of mTOR by rapamycin during priming and expansion of CD8^+^ T cells upon viral infection impairs the formation of Trm cells by blocking migration into the tissue, despite increasing the number of circulating memory T cells (41–43). This is consistent with data demonstrating that inhibition of mTOR induces Eomes and blocks persistent T-bet expression, favoring circulating memory T cell generation (44). These results suggest that differential modulation of mTOR-dependent early signals received during T cell activation can instruct circulating and memory compartments before tissue entry and differentiation. Moreover, cross-priming by type 1 classical DCs (cDC1s) is required for optimal generation of Trm but not circulating memory cells, supporting the notion that priming signals can imprint acquisition of a committed Trm cell fate (14). However, Trm precursors are not only derived from naive T cells, since antigen-experienced circulating memory T cells are also able to produce Trm cells after infection or in a tumor context (Box 1), highlighting the plasticity of the memory T cell subsets, as explained below. We will thus discuss the genealogy of CD8^+^ Trm cell generation and the differential role of DCs during priming, differentiation, and reactivation of Trm cells, highlighting them as a strategy in vaccination and tumor immunotherapies.

## Generation of Memory CD8^+^ T Cell Precursors

The traditional definition of memory T cells is based on the survival time after infection, once antigen-specific T cell numbers stabilize, which normally occurs several weeks to months after priming. However, this survival-based definition does not take into account some key functional aspects of memory T cells that, on the other hand, define diverse memory subsets. These characteristics comprise the capacity of memory cells to develop rapid recall responses, the high proliferative capacity or stemness, and the homeostatic turnover. We could hypothesize that CD8^+^ T cells do not acquire these memory-related functional features until infection has been controlled, meaning that effector cytotoxic T lymphocytes only become Trm-committed cells once they have been established in their destination tissue. Alternatively, divergent differentiation fates of T cell progeny could be specified when a naive T cell is activated during the acute phase of the immune response. Several evidences suggest that the fate of memory versus effector CD8^+^ T cells is determined early after priming or gradually during their development, meaning that memory cells are derived from early committed precursors (44–47). Notwithstanding, it is still not well understood whether this paradigm can be applied to Trm differentiation. The existence of an imprinted Trm precursor generated in secondary lymphoid organs is supported by the reconstitution of mature Trm cells upon KLRG1^−^ adoptive transfer (4). In this study, CD8^+^ effector cells isolated from spleen of gBT-I.1 transgenic mice expressing a TCR specific for the MHC class I-restricted immunodominant peptide from HSV glycoprotein B (gB498-505) were sorted 6 days after infection with HSV based on KLRG1 expression. The authors showed that, upon adoptive transfer, only the KLRG1^−^ population generated matured CD103^+^ Trm cells in the skin of HSV-infected recipient mice. Moreover, Trm differentiation requires a distinct program that combines effector and memory cell transcriptional programs, sharing some features with early effector CD8^+^ T cells or Tem cells but also some of the Tcm cell properties (39, 48).

**Figure 1:**
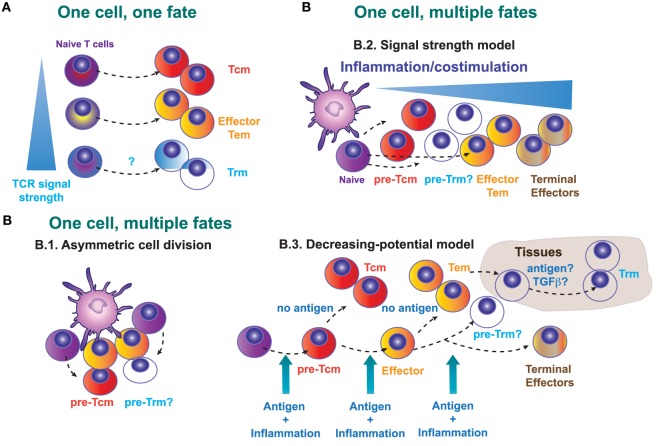
Possible models that explain the generation of a committed Trm precursor in secondary lymphoid organs. **(A)** One cell, one fate model. Distinct naive T cells will exhibit a different lineage decision determined by the quality (intensity of signal) of their TCR. **(B)** One cell, multiple fates model. B.1., Asymmetric cell division in T lymphocytes may determine fate diversification. B.2., Signal strength model. The strength of the signals 1, 2, and 3 determines the fate of the activated CD8^+^ T cells, with low strength signals generating central memory T (Tcm) precursors and high strength supporting the generation of terminal differentiated effectors. B.3., Decreasing potential model. This model proposes that a short duration of antigenic stimulation favors development of activated cells that will give rise to greater numbers of Tcm cells, while longer duration of stimulation promotes terminal effector cell differentiation and death.

There are several models that explain generation of committed precursors for Tcm, Tem, and Trm cells (Figure 1). The “one cell, one fate” model (Figure 1A) proposes that distinct fates emerge from different naive T cells, with one single activated T cell giving rise to daughters of only one fate. In other words, this “one cell, one fate” model suggests that naive T cells are predetermined during thymic development to give rise to effector or memory T cells. Therefore, we can speculate that specific TCR-bearing cells will give rise to circulating (Tcm or Tem) memory cells, while other CD8^+^ T clones expressing a different TCR will generate Trm cells. Nevertheless, Trm cell clones generated in the skin and Tcm cell clones in the draining lymph nodes (dLN) show a similar abundance of particular TCR clones tracked by CDR3 sequences, suggesting that a common naive T cell precursor is able to give rise to both Trm and Tcm cells after skin immunization (40). However, there may also be some pre-determination to become Trm or Tcm cells based on TCR-MHC interaction strength. For example, Trm cells in brain and kidney express TCRs with higher affinity to MHC-I tetramers (up to 20-fold) than their splenic memory T cells counterparts, whereas effector cells express similar high-affinity TCRs in all organs (49). Conversely, low-affinity T cells, with reduced T-bet expression during priming, preferentially differentiate into Tcm precursors (50). Similarly, different CD8 T cell clones have a distinct and fixed hierarchy in terms of effector function in response to the same *Toxoplasma* antigen measured as proliferation capacity, trafficking, T cell maintenance, and memory formation. Homing to the brain was directly related to TCR affinity. The highest affinity clone persisted longer in the host during chronic infection as a resident memory population (CD103^+^) in the brain (51). These data suggest that the non-lymphoid microenvironment may facilitate the retention of T cells with high-affinity TCRs, particularly in persistent infections, which would facilitate detection of infected cells expressing low levels of antigen. We can thus conclude that although the “one cell, one fate” model does not always explain how a naive CD8^+^ T cell become a Trm or a circulating memory cell, the clonal TCR affinity may influence on this Trm cell fate or their persistence, depending on the nature of the infectious pathogen, or the infected target tissue where Trm cells establish.

Alternatively, it is possible that effector T cells and different memory T cell subsets can derive from a single naive T cell clone (Figure 1B). This “one cell, multiple fates” model, proposes that the fate decision is taken during T cell priming or even in later stages during the T cell response. Several possible mechanisms may explain how different memory and effector subsets emerge from one single cell. During the immunological synapse between the antigen-presenting cell and the T cell, asymmetric cell division (Figure 1B.1) allows the generation of two different daughter cells. Accordingly, the generation of effector and memory T cells from naive T cells in primary responses could depend on the asymmetric inheritance of intracellular fate determinants (52). However, the relevance of this asymmetric cell division in the generation of different memory precursors has not been determined yet.

*In vivo* cell tracking of individual OT-I cells demonstrated that, even for T cells with the same TCR, there are heterogeneous patterns of clonal expansion and differentiation. Therefore, the dynamics of the single-cell response are not uniform, as demonstrated by the differential participation of their progeny during primary versus recall infections. Therefore, individual naive T lymphocytes contributed differentially to short- and long-term protection (53, 54). In addition, the progeny of naive clonal CD8^+^ T cells displayed unique profiles of differentiation based on extrinsic antiviral- or antibacterial-induced environmental cues. A single naive CD8^+^ T cell exhibited distinct fates that were controlled by tissue-specific events (55, 56). Following oral infection with *Listeria monocytogenes*, an antigen-specific CD8 T cell population can be separated into cells with a memory precursor phenotype in the intestine, whereas in the spleen and lung, *L. monocytogenes*-specific CD8 T cells maintained a prolonged short-lived effector phenotype. This intestinal CD127^+^ KLRG1^−^ CD8 T cell population resembling memory precursor formed in response to TGF-β following oral *L. monocytogenes* infection. This subset rapidly upregulated CD103 needed for association to the epithelium and survived long-term, identifying mucosal Trm precursors (56). In either case, these observations exclude models in which each naïve T cell exclusively yields progeny with the same distribution of either short- or long-term potential phenotype, arguing against asymmetric division as a singular driver of CD8^+^ T cell heterogeneity.

During priming, T cells receive three key signals: antigen recognition (signal 1), co-stimulation (signal 2), and cytokines that modulate T cell differentiation (signal 3). According to the “Signal strength model” (Figure 1B.2), the strength of the three signals will determine the expansion amplitude and the fate of the primed T cell (57). Generation of short-lived or terminally differentiated CD8^+^ T cells is favored by a strong pro-inflammatory signal (58), whereas precursors for Tcm cells are increased by the deficiency in type I interferon signaling (59), or deficiency in IFN-γ or IL-12 (60). Contrary to Tcm generation, inflammation drives Trm differentiation in several non-lymphoid tissues (9). Many tissue-specific cytokines including IL-15, TGF-β, IL-12, and type I IFN are produced upon infection and inflammation and regulate differentiation and persistence of Trm cells in non-lymphoid tissues, with differential requirements that may be tissue specific (4, 61, 62).

The “decreasing potential model” (Figure 1B.3) states that the history and accumulative duration of signals that a CD8^+^ T cell has encountered during infection impacts on its differentiation state. Repetitive antigen encounter and/or exposure to inflammatory cytokines, differentiates T cells toward terminal effector T cells that retain their cytolytic capacity but lose features owned by Tcm cells, such as longevity, proliferative potential, and IL-7Rα expression. In this sense, and contrary to Tcm cells, local antigen presentation may favor the expansion of Trm cells in the skin (14, 63). The composition of the local Trm cell pool is shaped by antigen-dependent competition between CD8^+^ T cells of different specificities in the infected tissue (64). Therefore, Trm cells development seems to be favored by antigen encounter and/or specific inflammatory signals in the tissue that favor, or are even needed for their retention (4, 9, 65). Regardless of the apparently contradictory different mechanisms proposed by these models, they are not necessarily mutually exclusive and multiple models may simultaneously contribute to *in vivo* induction of memory T cells.

## Plasticity among Different T Cell Subsets

**Figure 2:**
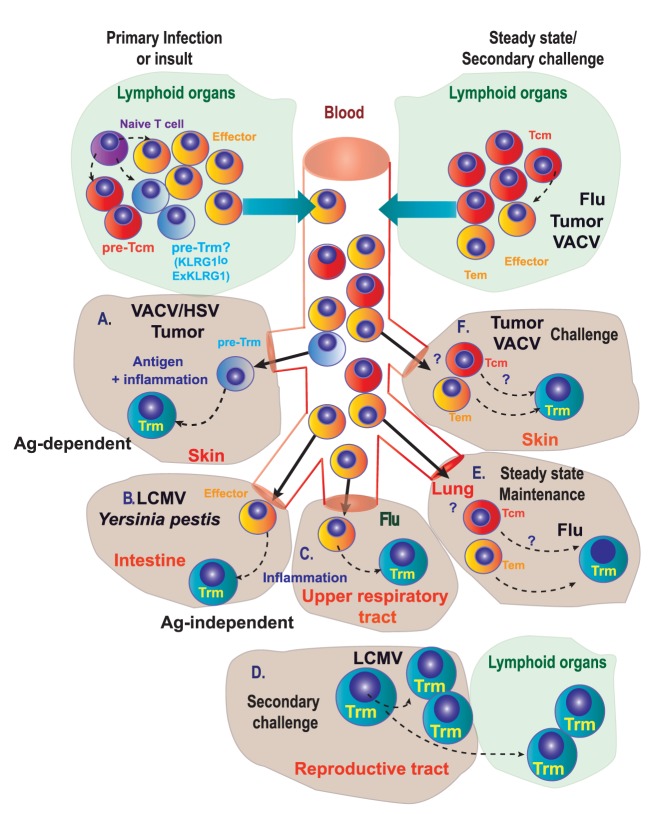
Genealogy of Trm. **(A)** During primary infection, immunization, or other insults, naive T lymphocytes differentiate into precursors of circulating memory cells, effectors, and putative precursors of Trm (pre-Trm) cells that can differentiate into Trm cells in the skin, in response to viruses (VACV/HSV) or tumors. **(B,C)** Inflammation in the intestine **(B)** or in the upper respiratory tract **(C)** is able to promote Trm generation. **(D)** In the female reproductive tract, proliferating pre-existing Trm cells contribute substantially to the boosted secondary Trm population and can exit non-lymphoid tissues to convert into new Trm cells in lymphoid tissues. **(E)** Under steady-state conditions, circulating memory T cells can differentiate into Trm cells in the lung of mice previously infected with influenza A virus. **(F)** Circulating memory [effector memory T (Tem) or central memory T (Tcm)] cells can differentiate into Trm cells in the skin upon secondary challenge with viruses or tumors.

Independently of the existence or not of a committed Trm precursor, it is well documented that naive (CD8^+^CD44^−^CD62L^+^) T cells differentiate into Trm cells in multiple scenarios: skin infection with VACV (3), or HSV (66), intranasal infection with influenza (67) or in non-infectious disorders, such as chemical hapten inflammation (40). In several cases, optimal generation of committed Trm precursors requires further antigen presentation in the inflamed tissue (Figure 2A). However, Trm differentiation and maintenance is dependent on tissue-specific signals that may be antigen independent. Inflammation drives Trm differentiation in many non-lymphoid tissues (9) (Figure 2B). Many tissue-specific cytokines including IL-15, TGF-β, IL-12, and type I IFN are produced upon infection and inflammation and regulate differentiation and persistence of Trm cells in non-lymphoid tissue, with differential requirements that may be tissue specific (4, 61, 62). Effector CD8^+^ T cells can also differentiate into nasal Trm cells independently of local antigen (68) (Figure 2C). However, it is difficult to know if the conversion of effector T cells into Trm occurs in all effector cells infiltrating the tissues, or whether there are specific features in the effector T cells that commit them to Trm differentiation under the right tissue environment, as we have discussed in the former section.

In steady state or upon challenge, Trm cells may also be generated from antigen-experienced cells: Tcm, Tem, and Trm cells themselves (self-maintenance) (65, 69–71). Local antigen reactivation of pre-existing Trm in the female reproductive tract (70) or the skin (69) results in their arrest and *in situ* division (Figure 2D). These proliferating Trm also exhibit some plasticity and can exit non-lymphoid tissues to convert into new Trm in the draining lymphoid tissue (71, 72) (Figure 2D). Although local mucosal recall response is dominated by proliferating pre-existing Trm that contribute most substantially to the boosted secondary Trm population, Trm reactivation also induces the antigen-independent recruitment of Tcm that differentiate into Trm *in situ* (69, 70). Maintenance of a Trm pool in the lung by conversion of incoming circulating memory CD8^+^ T cells is critical for protection after influenza A virus infection (73). Lung Trm cells are replenished mainly from circulating CD8^+^CD69^−^CD103^−^ Tem rather than Tcm cells, even in the absence of persisting antigen (Figure 2E). However, this lung Trm pool declines with time as circulating memory CD8^+^ T cells lose migratory capacity to the lung, together with an enrichment of Tcm versus Tem among circulating population of memory cells, thus reducing the efficiency of conversion to Trm cells. These findings support a model where gradual loss of protection to influenza is linked to a decline of Trm cells in the lungs caused by apoptosis and decreased input from the circulating memory CD8^+^ T cell population (73).

Transfer of CD8^+^CD44^+^CD62L^+^ Tcm cells specific for ovalbumin (OT-I) followed by epicutaneous VACV-OVA infection also induced Trm cells in the skin (12) (Figure 2F). The efficiency of Trm generation is, however, not equal depending on the different T cell source. For example, although both Tcm and naive T cells induce Trm cells that persist at least 2 months after infection, Tcm cells are less efficient at producing Trm cells (12). Most Trm cells generated from adoptively transferred Tcm cells showed hallmark CD69 expression, with half of them co-expressing CD103. Trm cells derived from adoptively transferred Tcm cells were unable to migrate *via* blood or lymph (12), supporting that they are *bona fide* Trm cells without recirculating capacity (74). Plasticity of transferred Tcm to become Trm cells does not only occur upon infection but also in the context of tumor challenge. Mice transferred with OVA-specific Tcm cells and challenged with intradermal inoculation of B16-OVA melanoma developed cells with a Trm cell phenotype (CD69^+^ CD103^+^) within the tumor mass. Furthermore, when mice transferred with Tcm cells were challenged with MC38-OVA colon adenocarcinoma, CD69^+^CD103^+^ OVA-specific CD8^+^ T cells were found in the skin proximal to rejected MC38-OVA tumors 45 days after inoculation (Box 1) (12). However, whether the conversion of Tcm into Trm occurs directly or is mediated by Tcm conversion into effector or Tem needs to be further studied.

## DCs Drive Trm Cell Priming and Reactivation

**Figure 3:**
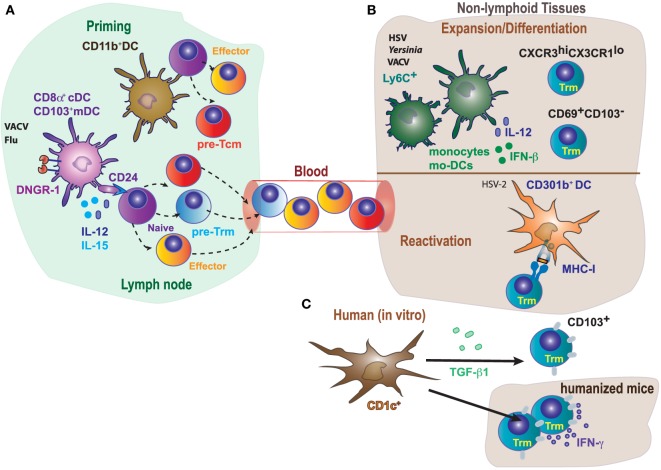
Differential role of antigen-presenting cells in priming, expansion/differentiation, and reactivation of tissue-resident memory T cells. **(A)** In mice, optimal generation of Trm in response to VACV or Flu requires CD8^+^ T cells cross-priming by DNGR-1^+^ dendritic cells (cDC1, CD8α^+^, and CD103^+^), while circulating memory T cells could be primed by both CD11b^hi^ (cDC2) or cDC1. Naive T cells cross-primed by cDC1 receive CD24 co-stimulation, IL-15 and IL-12 specifically produced by this dendritic cell (DC) subset, contributing to the generation of committed Trm precursors. **(B)** Monocytes (Ly6C^+^) and monocyte-derived DC (Mo-DCs) contribute to expansion of Trm in response to HSV or *Yersinia* by secretion of inflammatory cytokines, and also modulate the generation/differentiation of specific Trm subpopulations (CXCR3^hi^CX3CR1^lo^; CD69^+^CD103^−^). In addition, reactivation of Trm cells in response to HSV-2, requires MHC-I expression in CD301b^+^ DC. **(C)** Notably, human CD1c^+^, but not CD141^+^, induce CD103 expression on CD8^+^ T cells and their accumulation in the lung, in a process dependent on TGF-β1.

While most of the studies in Trm generation and development have focused on differentiation and maintenance dependent on specific tissue-derived signals, priming of committed precursors in the secondary lymphoid organs has been less explored. The analysis of mice deficient in DNGR-1 or Batf3 (75, 76) has shown the relevance of cDC1 in priming of CD8^+^ T cell memory subsets. High expression of DNGR-1 is restricted to the cDC1 subset, where DNGR-1 plays an essential role in cross-presentation to VACV antigens (77, 78). In addition, the cDC1 subset depends on the Batf3 transcription factor for their development and/or function (76, 79). Deficient cross-presentation by cDC1 results in a threefold reduction in the numbers of Trm cells in a model of skin VACV infection, while circulating memory CD8^+^ T cells are not affected (14) (Figure 3A). The cDC1 subset provides the antigen for priming by cross-presentation in this context of infection, but also provide specific signals 2 (CD24) and 3 (IL-12 and IL-15) (47, 80–83). These specific priming signals from cDC1s are also essential for optimal priming of Trm precursors (14), suggesting that priming by cDC1s is key for optimal Trm cell priming in this context of VACV infection, and cross-priming is the operational manner in which the antigen is presented in this setting. The key role of cDC1 for priming of Trm cells could be extended to additional infection models: for example, targeting malaria antigen to DNGR-1-expressing cDC1s in the presence of adjuvant generates Trm cells in the liver upon trapping primed T cells with a recombinant adeno-associated virus that targets hepatocytes to express the same malaria antigen (84).

Following viral infection, cross-priming transiently induces T-bet and its target CXCR3 in CD8^+^ T lymphocytes in the dLN, correlating with the generation of Trm precursors (14). T-bet induction at priming may contribute to longer retention in the LN of T cells that eventually egress to the tissue with low expression of T-bet and KLRG1. Consistent with the notion that high expression of T-bet inhibits Trm differentiation in the skin (36, 85), cross-priming ultimately favors T cells with delayed egress and lower expression of T-bet and KLRG1 in the skin (14). In addition, cross-priming transiently phosphorylates Foxo1 in CD8^+^ T cells (14), resulting in its degradation that favors retention of CD8^+^ T cells in the LN. However, cross-priming deficiency does not affect expression of the transcription factor Eomes, involved in Tcm generation (44, 58). Thus, this early transcriptional regulation by cross-priming does not affect effector or circulating memory CD8^+^ T cell development, IFN-γ production, or viral clearance mediated by CD8^+^ T cells. However, the analysis of CD103^+^ Trm cell differentiation in the skin revealed that formation of CD103^+^CD8^+^ T cells was slower between 7 and 14 days in the absence of cross-priming, suggesting a lower number of Trm cell precursors seeding the skin. Impaired Trm but not Tcm cell generation in vaccinated DNGR-1-deficient mice results in defective viral clearance (14).

Cross-priming through cDC1 also results in more prolonged downregulation of KLF2 and S1P (14). Downregulation of the KLF2-dependent S1P receptor leads to retention during priming (86). Weak priming in the absence of cross-presentation by cDC1s leads to early upregulation of KLF2 and S1P, leading to early egress of KLRG1^+^ cells that are not Trm precursors (4, 14) and migrate to the skin to generate terminal effector CD8^+^ T cells (58, 87). Once in the skin, inflammatory signals downregulate again KLF2 and S1P contributing to retention (23). Consistently, the inhibition of T cell egress with FTY720 treatment increases generation of both circulating memory and Trm cells in WT mice, partially rescuing the defect in Trm cell generation in mice deficient in cross-priming by cDC1s (14). These data highlight that retention of CD8^+^ T cells during priming in the LN favors Trm cell generation. However, it is not sufficient to compensate the specific signals provided by *Batf3*-dependent DNGR-1^+^ DCs. *In vitro* co-culture of CD8^+^ T cell with different DC subsets shows that CD103^+^ and CD8α^+^ DC (cDC1s) but not CD11b^hi^ CD8α^−^ (cDC2s) induce generation of Trm cells, in a DNGR-1-dependent fashion. The blockade of specific priming signals provided by cDC1s such as CD24, IL-12, and IL-15 reduces T-bet induction and generation of Trm precursors; however, cDC1 priming blockade does not affect the generation of circulating memory T cells (14).

It is debated to which extent antigen presentation (signal 1), co-stimulation (signal 2), or cytokines (signal 3) derived from different DC subsets are required for differentiation and for reactivation upon rechallenge. The requirement of antigen for Trm cell differentiation in tissues has been described (3, 14, 66, 67). Antigen recognition within the tissue drives expression of CD103 by brain Trm cells (8). The restimulation of Trm cells and induction of IFN-γ is dependent on MHC-I expression on CD301b^+^ DC (Figure 3B), while inflammatory cytokines alone are likely not sufficient by themselves for full activation of Trm cells responding to genital HSV-2 infection (88). However, antigen presentation is dispensable for Trm generation in other systems (4, 9, 89), supporting the notion that the particular pathogen or inflammatory insult triggers a distinct response that determines the requirements for Trm differentiation (40). Inflammatory signals derived from myeloid cells can also impact in the Trm cell phenotype (Figure 3B). Recruitment of monocyte-derived DCs in the LNs is required for the activation of HSV-specific CD8^+^ Trm cells (66). Ly6C^+^ inflammatory monocytes contribute to the persistence, but not generation, of lung memory CD8^+^ Trm cells, affecting selectively to a CXCR3^hi^CX3CR1^lo^ subset upon VACV intranasal challenge (90). Moreover, IFN-β and IL-12 derived from monocyte-derived intestinal macrophages during *Yersinia* infection, favors the differentiation of CD69^+^CD103^−^ Trm cells (Figure 3B) (62).

While cDC1s are essential for optimal priming, they are dispensable for differentiation in the skin, which also requires antigen presentation in the VACV infection model (14). Thus, different DC subsets may work cooperatively in the LN priming of Trm precursors and differentiation in the skin in an antigen-cognate fashion. However, the requirement of antigen presentation by different DC subsets may be model dependent. XCR1^+^ cDC1 seem to be necessary to promote recall of circulating memory CD8^+^ T cells upon secondary infections with pathogens such as *L. monocytogenes* or certain viruses (91), or in response to tumors (12). But this particular DC subset does not seem to play a role in the maintenance of Trm cells upon viral infection (14). In a mouse model of HSV-2 intravaginal infection, depletion of CD301b^+^ cDC2 results in significantly worse clinical symptoms, higher weight loss, and mortality after viral rechallenge (88). However, CD301b^+^ cDC2 depletion does not affect circulating memory, while stimulates the differentiation and antiviral function of vaginal CD8^+^ Trm cells (Figure 3B). Accordingly, CD301b^+^ cDC2 depletion has minimal impact on disease severity and weight loss when protection is exclusively dependent on circulating memory CD8^+^ T cells (88).

In comparison to murine DCs, less is known about the function of human DCs in tissues. By using lung tissues from humans and humanized mice, it has been found that both lung DC subsets (CD1c^+^ and CD141^+^) acquire antigens from live-attenuated influenza virus *in vivo* and expanded specific cytotoxic CD8^+^ T cells *in vitro* (Figure 3C). However, lung tissue-resident CD1c^+^ DC but not CD141^+^ DC induce CD103 expression on CD8^+^ T cells and promoted CD8^+^ T cell accumulation in lung. Induction of CD103 expression mediated by CD1c^+^ DCs was dependent on TGF-β1. Thus, CD1c^+^ and CD141^+^ DCs generate CD8^+^ T cells with different properties (92). The results discussed above are consistent with the notion of division of tasks among DC subsets during the priming and differentiation of Trm cells, although the particular role of a DC subset or even the dependence on antigen presentation or priming by DC-derived cytokines may depend on the particular settings in which Trm cells are generated.

## Concluding Remarks

Following immunization, DCs in the secondary lymphoid organs prime CD8^+^ T cells for generation of effector and memory responses, but there are different flavors of memory T cells and the specific requirements for priming, differentiation, and reactivation of each subset are different. Tissue-resident memory CD8^+^ T (Trm) cells represent the newest layer of complexity in memory subsets. By virtue of their location, they act as sensor and effector cells, triggering both innate and adaptive responses, therefore providing a superior immunity against reinfection in the tissue (3, 4, 93).

Current evidences support the idea that Tcm, Tem, and Trm cells are generated from common precursors that are committed upon differential priming in secondary lymphoid organs (14, 40). Asymmetric inheritance of intracellular fate determinants could explain generation of effector and memory T cells from clonal naive T cells (52). TCR affinity and duration of signals during priming can also determine the T cell fate, with Trm cells linked to high-affinity TCR and Tcm cells to low affinity (49, 94).

Naive T cells differentiate into Trm in many scenarios: infectious and even non-infectious, such as chemical hapten inflammation (3, 12, 40, 66, 67). However, independently of the generation of Trm cells from naive cells primed in the LN, there is some degree of plasticity among T cell subsets. Trm cells can be generated from antigen-experienced cells such as effector CD8^+^ T cells, Tcm, Tem, or even Trm cells (self-maintenance). Several factors may condition the relative efficiency of Trm generation from difference sources, including the type of challenge (infection, inflammation), the presence of specific antigen driving reactivation and tissue-specific signals that can promote Trm generation in an antigen-independent fashion (12, 70, 71, 73).

Different subsets of DCs may affect differentially the priming of Trm precursors. cDC1s drive priming of Trm precursors in the LN, but not Trm tissue differentiation, in a VACV skin infection model, and targeting malaria antigen to cDC1s generates antigen-specific Trm in the liver, requiring both models antigen presence in the target tissue (14, 84). Antigen presentation and inflammatory cytokines produced by other myeloid cell subsets contribute to Trm differentiation (62, 88, 90, 92), suggesting a division of tasks among DC subsets in the priming and differentiation of memory T cell subsets that can be model dependent. Further dissection of how DC prime and generate different memory T cell subsets, what are the requirements for differentiation and effector function of each subset, and how these memory T cell subsets act in concert to induce optimal immunity will be important to improve current immunotherapy strategies against pathogens or cancer.

## Author Contributions

ME, SK, SI, and DS conceived and wrote the manuscript. ME and SI did the figures.

## Conflict of Interest Statement

The authors declare that the research was conducted in the absence of any commercial or financial relationships that could be construed as a potential conflict of interest.
